# Determinants of Improved Dyspnea and Exercise Tolerance With Nasal High‐Flow O_2_ Therapy in Fibrotic Interstitial Lung Disease: A Pilot Physiological Study

**DOI:** 10.1002/resp.70251

**Published:** 2026-04-09

**Authors:** Sarah Thivent, Marylise Ginoux, Samuel Verges, Frédéric Hérengt, Mathieu Marillier

**Affiliations:** ^1^ Cardiopulmonary Rehabilitation Centre Dieulefit Santé Dieulefit France; ^2^ HP2 Laboratory, INSERM U1300, CHU Grenoble Alpes Univ. Grenoble Alpes Grenoble France

**Keywords:** dyspnea, exercise, hypoxia, muscle fatigue, oxygen inhalation therapy, pulmonary fibrosis

## Abstract

**Background and Objective:**

Severe hypoxemia, heightened dyspnea, and exercise limitation are hallmarks of fibrotic interstitial lung disease (*f*‐ILD). Standard O_2_ therapy (nasal prongs) fails, however, to correct hypoxemia with limited symptomatic benefits due to inspiratory flow‐patient demand mismatch. Nasal high‐flow O_2_ therapy (NHFO_2_) is a promising alternative, but the respective contribution of high‐flow and supplemental O_2_ to improved dyspnea and exercise tolerance remains unknown in *f*‐ILD.

**Methods:**

Sixteen patients performed, in a randomized order, endurance tests (70% peak power) under 4 conditions: air, supplemental O_2_ (face mask, 9–12 L·min^−1^), NHF_air_ [50–70 L·min^−1^; inspired fraction of O_2_ (FiO_2_) = 0.21], NHFO_2_ (50–70 L·min^−1^; FiO_2_ = 0.5). Endurance time and O_2_ saturation (SpO_2_), breathing pattern (respiratory plethysmography) and dyspnea (Borg CR‐10) were compared across conditions.

**Results:**

Supplemental O_2_ (98 [2]%) and NHFO_2_ (99 [3]%) increased isotime SpO_2_ vs air (87 [17]%, *p* < 0.001). Exercise time improved on O_2_ and NHFO_2_ vs air and NHF_air_ (683[903], 690[1338], 346[247], 319[415]s, respectively, *p* < 0.001; O_2_ vs NHFO_2_, *p* = 0.117). Supplemental O_2_ and NHFO_2_ reduced isotime ventilation vs air (47 ± 22, 44 ± 20, 63 ± 29 L·min^−1^, *p* < 0.001), driven by lower respiratory rates (36 ± 9, 37 ± 8, 44 ± 10 br·min^−1^, *p* < 0.001). Supplemental O_2_ and NHFO_2_ reduced isotime dyspnea vs air (4[3.5], 3.5[2.5], 7 [3], *p* < 0.001) and NHFO_2_ vs NHF_air_ (3.5[2.5] vs 6[1.5], *p* = 0.016). NHF_air_ lowered isotime ventilation (9.0 ± 6.2 L·min^−1^, *p* = 0.012) but did not improve dyspnea and exercise time vs air.

**Conclusion:**

Supplemental O_2_ and NHFO_2_ improved dyspnea and exercise time at “iso‐O_2_ saturation” in severely hypoxemic *f*‐ILD. Physiological benefits from supplemental O_2_ (including lower ventilation) are thus likely primary drivers of dyspnea relief and improved exercise tolerance on NHFO_2_ vs air in *f*‐ILD.

**Clinical Trial Registration:**

NCT07129707 (registered at ClinicalTrials.gov).

We tested the respective effect of high‐flow and supplemental O_2_ from nasal high‐flow O_2_ therapy (NHFO_2_) on dyspnea and exercise tolerance in fibrotic interstitial lung disease. Supplemental O_2_ and NHFO_2_ (but not high‐flow) provided improvements in these outcomes at “iso‐O_2_ saturation” due to reduced ventilatory requirements. Physiological benefits derived from O_2_ supplementation are thus likely primary drivers of dyspnea relief and improved exercise tolerance on NHFO_2_ (vs air) in these patients.

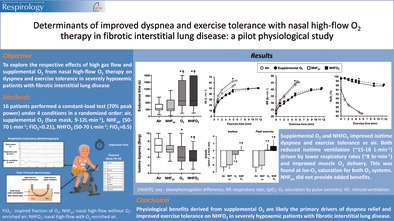

## 1 Introduction

Fibrotic interstitial lung disease (*f‐*ILD) encompasses a diverse group of over 200 restrictive lung conditions, primarily characterized by scarring of the lung parenchyma and variable degrees of inflammation and fibrosis [1, 2]. Key features of *f‐*ILD include reduced lung compliance, oxygen (O_2_) diffusion limitation and ventilation‐perfusion mismatch, the last two conspiring to impair gas exchange efficiency [3]. Consequently, patients commonly experience severe hypoxemia and heightened activity‐related dyspnea [4, 5], which substantially contribute to limiting their tolerance to physical exertion [3].

Ambulatory O_2_ therapy is often prescribed in *f*‐ILD to reverse or, at least, mitigate severe hypoxemia, improving dyspnea and exercise capacity being two critical outcomes for these patients [6]. Different laboratory‐based studies highlighted the benefits of supplemental O_2_ during exercise in *f‐*ILD; this includes reduced inspiratory neural drive and ventilation leading to dyspnea relief, but also improved muscle O_2_ delivery and fatigue, overall enhancing exercise tolerance [7, 8, 9]. Such laboratory findings reporting marked benefits, however, lack external validity as they cannot be easily translated into clinical practice due to the gas delivery system used (a Douglas reservoir bag connected to a two‐way non‐rebreathing valve being extremely effective at preventing arterial O_2_ desaturation) [10].

In this context, the efficacy of conventional O_2_ therapy (nasal prongs) is tempered by notable limitations in *f‐*ILD, with accumulating studies showing no or limited symptomatic or physiological benefits ([11, 12, 13], reviewed in Reference [6]). In fact, standard O_2_ delivery systems may not provide sufficient O_2_ flow to meet the heightened demand on exertion [14], failing to match peak inspiratory flow required by patients under physical strain [15]. Such mismatch leads patients to inhale ambient air thus diluting the inspired fraction of O_2_ (FiO_2_) [16] and resulting in incomplete correction of hypoxemia in *f*‐ILD [13]. In addition, the use of dry, unconditioned O_2_ poses further challenges: without adequate humidification, patients may experience nasal dryness, crusting, and mucosal irritation, the net result being reduced tolerance for therapy [17]. Exposure to cold, dry air may also trigger a bronchoconstrictive reflex, increasing airway resistance [18, 19].

Nasal high‐flow O_2_ (NHFO_2_) therapy is an easy‐to‐use gas delivery system supplying heated and humidified O_2_‐enriched air allowing the accurate monitoring of FiO_2_ at flow rates up to 70 L·min^−1^ [20]. This system has emerged as a promising alternative in *f*‐ILD to overcome current pre‐specified limitations: NHFO_2_ is more effective in reversing hypoxemia and reducing dyspnea vs standard O_2_ therapy (nasal prongs) [13] and consistently improved exercise capacity vs room air across studies in this patient population [13, 20, 21]. Yet, whether NHFO_2_ offers superior benefits vs O_2_ therapy supplied via a Venturi mask remains unclear, with either comparable [21] or greater [20, 22] exercise tolerance under the former. Nasal high flow (without O_2_‐enriched air, NHF_air_) exerts several physiological benefits that may be particularly relevant for patients with *f‐*ILD; these include rapid washout of the anatomical dead space preventing rebreathing of expired gas [23] and reduced work of breathing [24], which overall lowers patients' respiratory rate and ventilation [25, 26] and improves gas exchange efficiency [23, 27]. Collectively, these premises suggest that NHF_air_ may be valuable for enhancing exercise tolerance in *f*‐ILD. The physiological mechanisms underlying the positive effects of NHFO_2_ therapy remain, however, poorly understood; specifically, the respective contribution of high gas flow delivered via nasal cannula and greater oxygenation (with supplemental O_2_) on improving exertional dyspnea and exercise tolerance has never been explored in this patient population. This interrogation may only be addressed if supplemental O_2_ and NHFO_2_ improved O_2_ saturation to a similar extent (i.e., “iso‐O_2_ saturation”) to tease out the contribution of NHF_air_ from O_2_ supplementation (in NHFO_2_) in improving these outcomes.

To address this gap in knowledge, this prospective, randomized‐controlled trial aimed to disentangle the (i) independent and (ii) combined effects of respiratory support and supplemental O_2_ on dyspnea and exercise tolerance in patients with *f*‐ILD. We hypothesized that (i) NHF_air_ (due to lower ventilation and improved gas exchange efficiency) and supplemental O_2_ (through lower ventilation and larger muscle O_2_ delivery) would lessen dyspnea and improve exercise tolerance vs room air and (ii) NHFO_2_ would yield additive and thus greater benefits on these outcomes than either condition alone (even when reasoning at “iso‐O_2_ saturation” between supplemental O_2_ and NHFO_2_).

## 2 Methods

### 2.1 Participants

This prospective, randomized‐controlled, cross‐over trial enrolled patients with a diagnosis of *f*‐ILD according to internationally established criteria [2, 28] between December 2023 and January 2025. They were admitted to Dieulefit Santé Cardiorespiratory Rehabilitation Center (France) and enrolled prior to inpatient pulmonary rehabilitation initiation. They were considered for study inclusion if: (1) age ≥ 18 years; (2) modified Medical Research Council scale ≥ 1; (3) stable condition (no acute exacerbation within the preceding 3 months). Exclusion criteria were: (1) inability to perform a cardiopulmonary exercise test (CPET) on a bicycle ergometer; (2) participation in a pulmonary rehabilitation program within the last 6 months. This study was approved by an independent ethics board (CPP SUD‐OUEST and OUTRE MER III, IDRCB: 2022‐A00774‐39) and registered at clinicaltrials.gov (NCT07129707). Written informed consent was obtained from all participants before study enrollment.

### 2.2 Study Design

**FIGURE 1:**
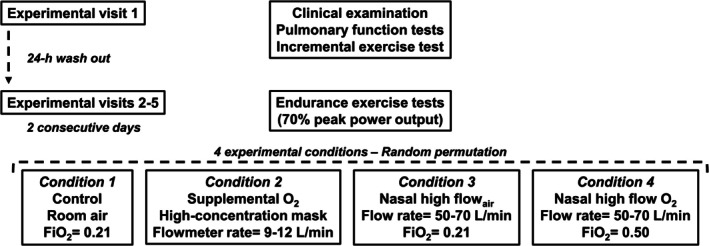
Outline of study design and experimental visits. After a first experimental visit (including, in particular, an incremental exercise test to determine peak power output) and a 24‐h wash out, participants performed 4 endurance exercise tests at 70% of peak power output to symptom limitation (experimental visits 2–5). Each of these tests was completed under a given experimental condition: Control (prespecified as *condition 1*), O_2_ supplementation (*condition 2*), nasal high‐flow without O_2_‐enriched air (NHF_air_, *condition 3*), and nasal high‐flow with O_2_‐enriched air (NHFO_2_, *condition 4*). Each participant was given a random permutation (over 24 potential ones) so that experimental conditions were performed in a randomized order. Endurance exercise tests were performed over 2 consecutive days; on each day, exercise testing took place at 11 am and 3 pm. See text for further elaboration. FiO_2_: Inspired fraction of oxygen; O_2_: Oxygen.

The outline of study design is depicted in Figure 1. Patients underwent a total of five visits. In the first visit, participants performed an incremental CPET on a bicycle ergometer (Ergoselect 4, Ergoline GmbH, Bitz, Germany) to determine room air peak power output (PPO). After a 1‐min resting period, power was increased by 10 W every minute until symptom limitation. Breath‐by‐breath metabolic and ventilatory analyses were obtained (Quark CPET, Cosmed, Pavona, Italy); Neder et al. [29] equations determined % predicted values for PPO and O_2_ uptake.

Participants completed four additional visits consisting of endurance exercise tests (EET, 70% PPO) at a self‐selected cadence ≥ 60 revolutions·min^−1^ until symptom limitation (or for a maximum of 30 min for practical purposes). After a 1‐min resting period and warm‐up of 1 min of unloaded cycling, power was increased to the desired intensity. Endurance exercise tests were conducted over two consecutive days; on each day, exercise testing took place at 11 am and 3 pm. These tests were performed under four experimental conditions: room air (prespecified as *condition 1*); supplemental O_2_ (*condition 2*); NHF_air_ (*condition 3*); NHFO_2_ (*condition 4*). Each participant was given a random permutation (over 24 potential ones) so that conditions were performed in a randomized order; each permutation was kept in a sealed envelope until the first EET.

### 2.3 Experimental Conditions

During the control condition, patients breathed room air (fraction of inspired O_2_ = 0.21) wearing no gas mixture delivery system. Supplemental O_2_ was provided via a non‐rebreather face mask; the flowmeter rate was selected to ensure O_2_ saturation remained **≥** 90% during exercise, using a fixed flow of 9–12 L·min^−1^ (Figure 1) to prevent carbon dioxide rebreathing and reservoir collapse. Nasal high flow (with or without O_2_) was provided through asymmetrical nasal cannula (Optiflow^+^ Duet, Fisher & Paykel, Auckland, New Zealand) by an AIRVO [3] system (Fisher & Paykel, Auckland, New Zealand). Supplied gas was heated to 37°C and fully humidified at 100%. Patients were first exposed to a flow rate of 30 L·min^−1^ at rest during 3 min (prior to starting exercise testing). Upon exercise start, it was individually adjusted to meet patients' ventilatory demand (i.e., the ventilation observed at the peak of incremental CPET) using a maximum of 70 L·min^−1^. We did not need to reduce flow rate in any patient after exercise start due to flow intolerance and thus used fixed values between 50 and 70 L·min^−1^ in each individual patient (Figure 1). Inspired fraction of O_2_ was set at 0.21 and 0.50 on NHF_air_ and NHFO_2_, respectively. Although participants were not blind to gas delivery systems as per the study objectives, they were blind to gas mixture and O_2_ saturation readings [30].

### 2.4 Measurements

#### 2.4.1 Rest

Participants completed full pulmonary function tests [spirometry, static lung volumes, lung diffusing capacity for carbon monoxide (MasterScreen Body, Erich Jaeger GmbH, Wuerzburg, Germany)] [31, 32, 33]. We assessed dyspnea with the modified Medical Research Council scale [34].

#### 2.4.2 Endurance Exercise Tests

##### 2.4.2.1 Time to Exercise Intolerance

Endurance time during EET (in s) was defined as the duration from exercise start to symptom limitation, excluding warm‐up. Patients were blind to ongoing exercise duration across experimental conditions. A 105‐s (95% CI = 60–140 s) or 33% (95% CI = 18%–48%) change between experimental conditions was deemed clinically meaningful [35].

##### 2.4.2.2 Cardiorespiratory and Gas Exchange Parameters

Heart rate was continuously monitored using a 12‐lead electrocardiogram (Quark T12x, Cosmed, Pavona, Italy). Oxygen saturation by pulse oximetry (SpO_2_) and transcutaneous partial pressure of carbon dioxide (tcPCO_2_) were measured by earlobe capnography (Sentec Digital Monitoring System, Sentec AG, Therwil, Switzerland). Minute ventilation (tidal volume × respiratory rate) was continuously assessed by respiratory inductive plethysmography (QDC Pro, Nox Medical, Reykjavik, Island). The system was calibrated using LabChart 8 (ADInstruments, Dunedin, New Zealand). A pneumotachograph was first calibrated with a 3L syringe, followed by an automatic calibration of the recording bands for 5 min. Then, a 90‐s calibration was performed with patients breathing into the pneumotachograph, lips closed around the mouthpiece, while wearing the bands. These steps allowed for the determination of a calibration factor to adjust volumes measured by the respiratory inductive plethysmography system. Data were filtered with a 10‐s smoothing filter before extraction.

##### 2.4.2.3 Near‐Infrared Spectroscopy

Muscle oxy‐ anddeoxyhemoglobin and oxy‐ and deoxymyoglobin concentrations ([O_2_Hb + Mb] and [HHb + Mb], respectively) were measured throughout sessions using a two‐wavelength (760 and 850 nm), spatially‐resolved near infrared spectroscopy (NIRS) system (Train. Red FYER, Elst, The Netherlands). The probe was positioned over the lower third of the *vastus lateralis* (~10–15 cm above the patella superior edge) [36]. [O_2_Hb + Mb] and [HHb + Mb] were measured as differences from the initial rest period during exercise. Total hemo‐ and myoglobin concentration ([tHb + Mb]) was calculated as [O_2_Hb + Mb] + [HHb + Mb]. Oxy‐deoxyhemoglobin and myoglobin concentration difference ([Hb + MbDiff] = [O_2_Hb + Mb] – [HHb + Mb]) was derived as an estimate of change in tissue oxygenation [37]. Data were recorded at 10 Hz and filtered with a 2‐s moving average filter.

##### 2.4.2.4 Perceptual Measurements

The intensity of dyspnea and leg discomfort were assessed using the 0–10 category‐ratio Borg scale [38] using standardized prompts “How intense is your sensation of breathing discomfort?” and “How intense is your sensation of leg discomfort?”; scores were obtained at rest, every 2 min throughout exercise and at exercise cessation. The Borg scale was anchored before exercise, that is, by explaining its endpoints (ranging from 0 to 10). A 1‐Borg unit change in isotime dyspnea between conditions was deemed clinically meaningful [35].

### 2.5 Data and Statistical Analyses

Cardiorespiratory and NIRS data were averaged over the last 30 s of resting periods, over a 30‐s epoch for “isotime” measurements (the time corresponding to exercise cessation in the shortest condition [39]) and over the last 30 s in each condition for peak‐exercise measures. We performed sample size estimate on G*Power 3.1.9.7 (Heinrich‐Heine‐Universität, Düsseldorf, Germany). Based on previous research from our group reporting an average improvement in endurance time of 360 ± 390% on NHFO_2_ vs room air (effect size = 0.92; from 171 ± 76 to 618 ± 297 s) during a EET [13], with α = 0.05 and a two‐tailed test of significance, a sample of 15 complete patients (i.e., able to complete study protocol with appropriate data collection for analysis) would provide a statistical power of 91%. Subsequent statistical procedures were performed on Statistica v.10 (Statsoft, Tulsa, OK, USA).

Normality of data distribution for continuous physiological variables was assessed with a Shapiro–Wilk test. For normally‐distributed data, we applied 1‐ or 2‐way repeated measures ANOVA (e.g., for NIRS data collected throughout EET: [experimental condition (room air, supplemental O_2_, NHF_air_, NHFO_2_) × exercise time (rest, isotime, peak exercise)]). Bonferroni post hoc tests were applied to adjust *p* values for multiple comparisons when ANOVA revealed a significant main or interaction effect. For non‐normally distributed data and discrete variables (i.e., symptoms), we used a Friedman ANOVA. Post hoc tests were Wilcoxon matched‐pairs if Friedman ANOVA reached significance. We used dependant‐sample Hodges‐Lehman Median Difference test for non‐normally distributed data and discrete variables to determine between‐condition differences and 95% confidence intervals. Findings were interpreted with the minimal clinically‐important difference (MCID) if available (i.e., exercise time and isotime dyspnea) [40]. Data are presented as mean ± SD for normally‐distributed continuous variables and median [interquartile range] for non normally‐distributed and discrete variables. A two‐tailed α level of 0.05 was used as the cut‐off for significance.

## 3 Results

### 3.1 Participants

**FIGURE 2:**
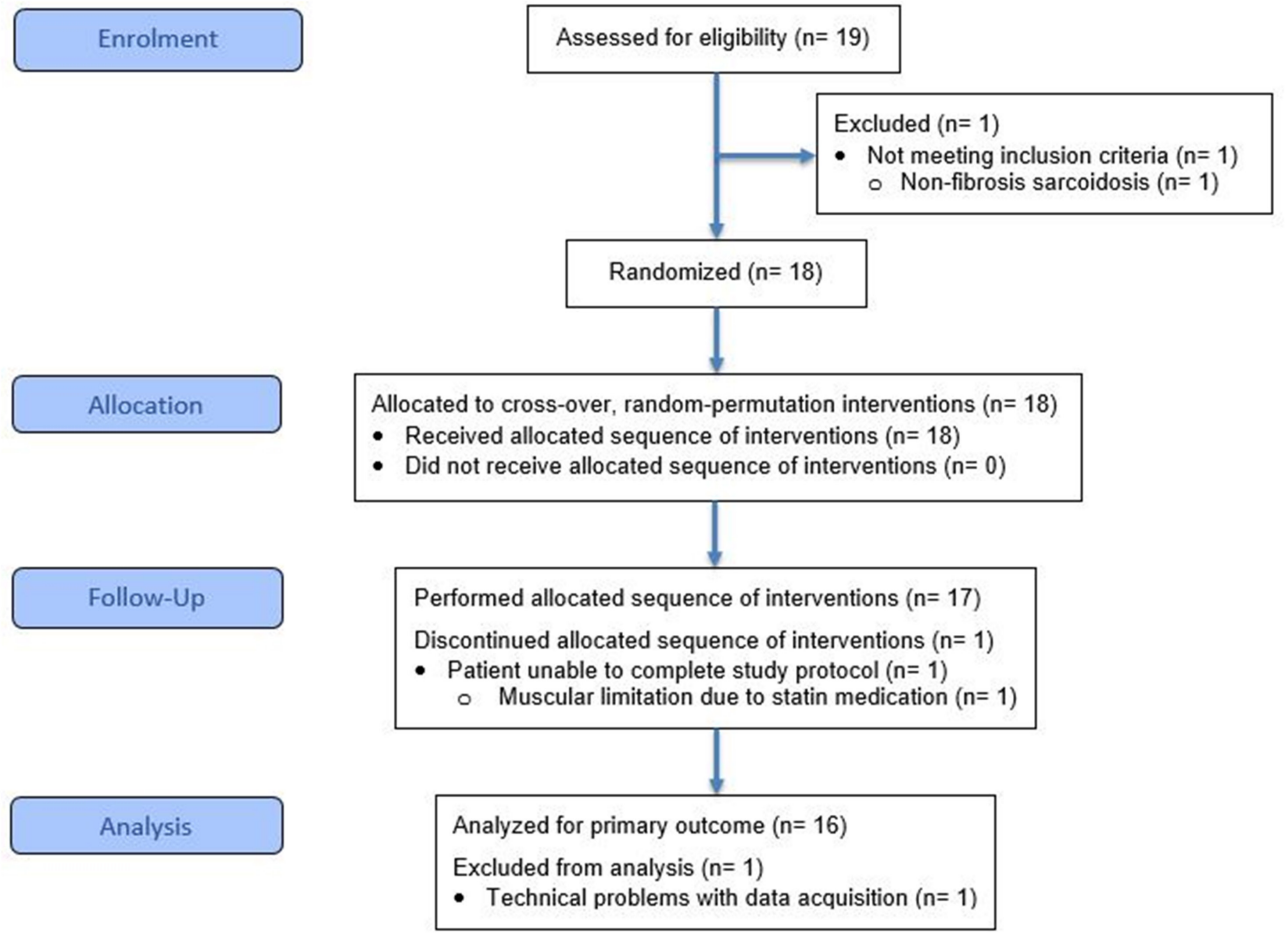
Flowchart of patients' enrolment.

Nineteen patients were assessed for study participation: after application of inclusion and exclusion criteria, 16 patients finished the study with complete data and were therefore included for analysis (Figure 2). Detailed characteristics of patients are provided in Table 1. Briefly, idiopathic pulmonary fibrosis was the most common aetiology of *f*‐ILD; 11 patients required daily ambulatory O_2_ therapy. Patients typically presented with low exercise tolerance (peak O_2_ uptake and power output = 85% ± 22% and 60% ± 27% predicted from incremental exercise testing, respectively). Of note, 14 patients (88% of sample) showed severe exercise‐related hypoxemia, that is, SpO_2_ ≤ 88%.

**TABLE 1:** Characteristics of patients with fibrotic interstitial lung disease.

	Patients (*n* = 16)
General characteristics	
Sex	9 ♂–7 ♀
Age (years)	68.9 ± 14.1
BMI (kg·m^−2^)	25.1 ± 3.8
Smoking history (pack·year^−1^)	0 [13]
mMRC scale score	2 [1]
Aetiology of *f*‐ILD	
Idiopathic pulmonaryfibrosis (*n*; %)	6 (38)
Scleroderma‐associated *f*‐ILD (*n*; %)	4 (25)
Non‐specific interstitial pneumonia (*n*; %)	3 (19)
Usual interstitial pneumonia (*n*; %)	1 (6)
Lymphoid interstitial pneumonia (*n*; %)	1 (6)
Unclassifiable *f*‐ILD (*n*; %)	1 (6)
Antifibrotic medications	
Nintedanib (*n*; %)	5 (31)
Pirfenidone (*n*; %)	1 (6)
Ambulatory oxygen therapy	
Use (*n*; %)	11 (69)
Pulmonary function	
FEV_1_ (L; % pred)	1.82 ± 0.66 (74 ± 27)
FVC (L; % pred)	2.27 ± 0.94 (71 ± 27)
FEV_1_/FVC (%; % pred)	82 ± 8 (105 ± 10)
TLC (L; % pred)	4.05 ± 1.51 (71 ± 24)
VC (L; % pred)	2.29 ± 0.95 (64 ± 24)
RV (L; % pred)	1.74 ± 0.62 (85 ± 32)
FRC (L; % pred)	2.31 ± 0.82 (70 ± 31)
DL_CO_ (mmol·min^−1^·kPa; % pred)*	2.33 ± 1.24 (31 ± 14)
Incremental exercise test	
Peak O_2_ uptake (L·min^−1^; % pred)	1.17 ± 0.45 (85 ± 22)
Peak O_2_ uptake (mL·kg·min^−1^)	16.8 ± 6.0
Peak power output (W; % pred)	63 ± 30 (60 ± 27)
Peak‐exercise SpO_2_ (%)	80 ± 7
Peak‐exercise SpO_2_ ≤ 88% (*n*; %)	14 (88)

*Note:* Data are mean ± SD or median [interquartile range].

Abbreviations: BMI: body‐mass index; DL_CO_: lung diffusing capacity for carbon monoxide; FEV_1_: forced expiratory volume in one second; *f*‐ILD: fibrotic interstitial lung disease; FRC: functional residual capacity; FVC: forced vital capacity; mMRC: modified medical research council; RV: residual volume; SpO_2_: oxygen saturation by pulse oximetry; TLC: total lung capacity; VC: vital capacity. * measurement adequately performed in 10 participants.

### 3.2 Main Outcomes—Exercise Tolerance and Isotime Dyspnea

**FIGURE 3:**
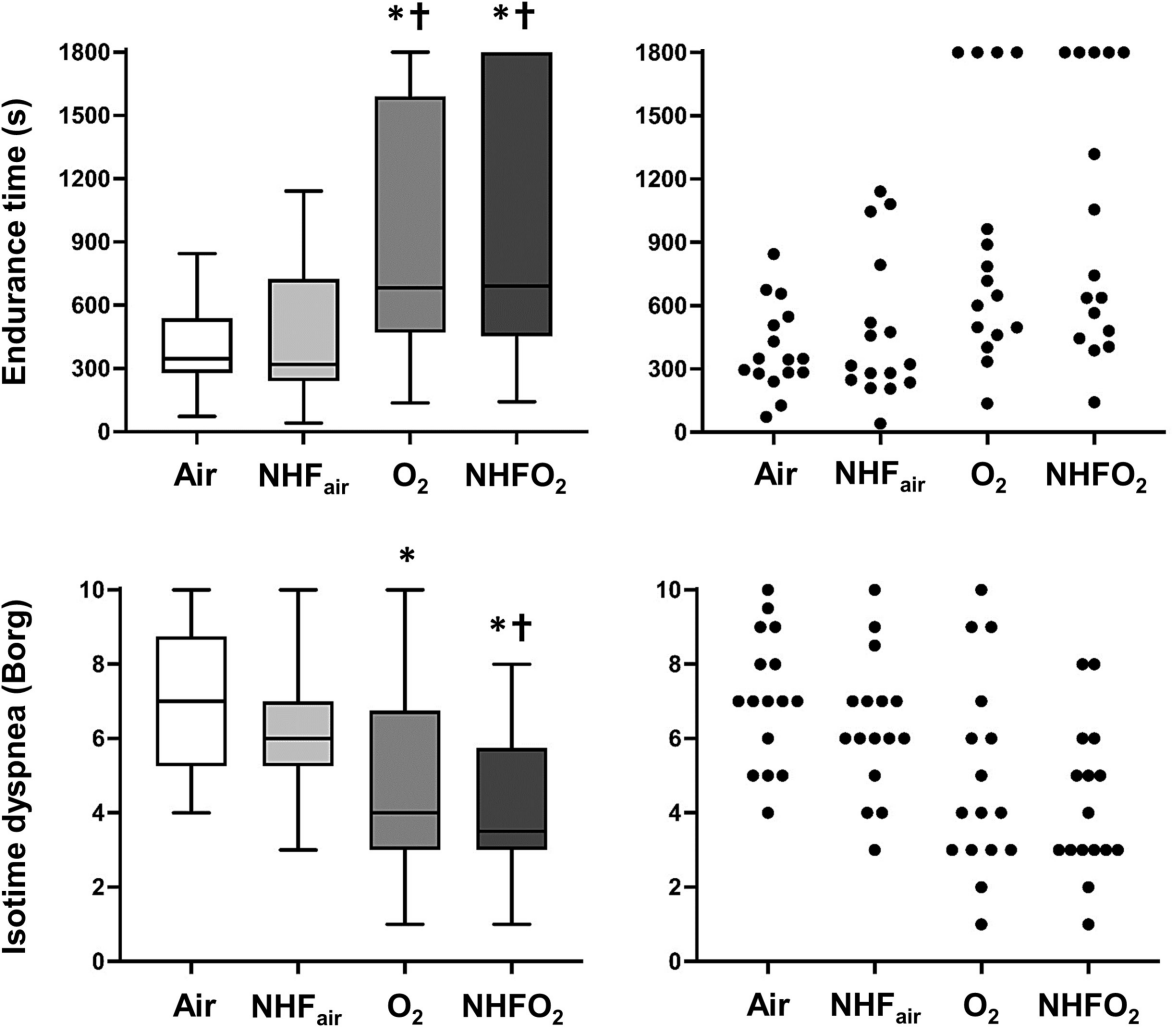
Time to exercise intolerance and “isotime” exertional dyspnea in patients with fibrotic interstitial lung disease across experimental conditions during endurance exercise test. Left and right panels respectively depict group and individual subject data. *p < 0.05 vs air; †: *p* < 0.05 vs NHF_air_ (post hoc analyses after Friedman ANOVA revealed a main effect of experimental condition, both *p* < 0.001). NHF_air_: Nasal high‐flow without O_2_‐enriched air; NHFO_2_: Nasal high‐flow with O_2_‐enriched air; O_2_: Oxygen supplementation.

Data for endurance time and isotime (289 [210] s) dyspnea across experimental conditions are depicted in Figure 3. Nine out of 16 patients (56%) had an endurance time during EET on room air between 180 and 480 s, thus falling within the “desirable” time limit range [35]. Four and 5 patients exercised for 30 min (upper limit set by the protocol) on O_2_ supplementation and NHFO_2_, respectively. Longer endurance time was found on supplemental O_2_ (683 [903] s) vs room air (346 [247] s, *p* < 0.001) and NHF_air_ (319 [415] s, *p* = 0.001); similar results were found for NHFO_2_ (690 [1338] s) vs room air and NHF_air_ (both *p* < 0.001). Overall, supplemental O_2_ and NHFO_2_ yielded worthwhile to very‐worthwhile effects on endurance time vs room air and NHF_air_ (lower bounds of 95% CIs>once to twice the MCID). Endurance time was not statistically different on room air vs NHF_air_ (*p* = 0.109) and on supplemental O_2_ vs NHFO_2_ (*p* = 0.117). Overall, NHF_air_ vs air and NHFO_2_ vs supplemental O_2_ yielded trivially‐small effect, though the estimate ranged from no to a worthwhile effect according to 95% CIs. Lower isotime dyspnea ratings were found on supplemental O_2_ (4 [3.5]) vs room air (7 [3], *p =* 0.001) but not NHF_air_ (6 [1.5], *p* = 0.071); NHFO_2_ lowered dyspnea (3.5 [2.5]) vs room air (*p* < 0.001) and NHF_air_ (*p* = 0.016). Supplemental O_2_ and NHFO_2_ had worthwhile to very‐worthwhile effects on isotime dyspnea vs room air and NHF_air_, though the estimate ranged from no to a worthwhile effect according to 95% CIs. Isotime dyspnea was not different on room air vs NHF_air_ (*p* = 0.147) and on supplemental O_2_ vs. NHFO_2_ (*p* = 0.117). NHF_air_ had trivially‐small effect vs room air and NHFO_2_ had worthwhile effect vs supplemental O_2_, though both estimates ranged from no to a worthwhile effect according to 95% CIs.

### 3.3 Cardiorespiratory, Gas Exchange and Perceptual Measurements

**FIGURE 4:**
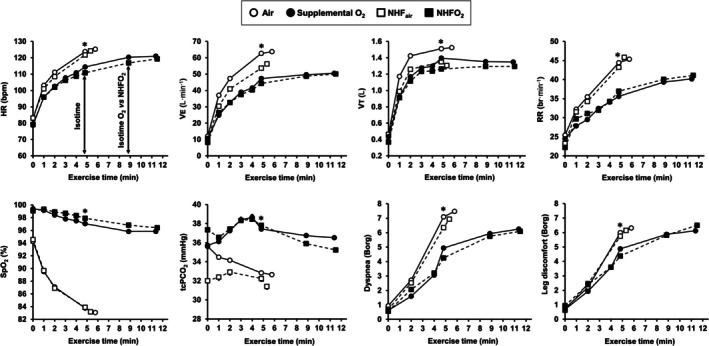
Physiological and perceptual responses in patients with fibrotic interstitial lung disease throughout endurance exercise tests across experimental conditions. * See Table 2 for significant differences and Table 3 for between‐condition differences and 95% confidence intervals across experimental conditions at a standardized time (isotime) during exercise. Data are shown at rest, 1 and 2 min in room air, supplemental O_2_, NHF_air_ and NHFO_2_, and at 3 and 4 min in supplemental O_2_ and NHFO_2_. Data are also depicted at isotime (median time = 289 s), isotime between supplemental O_2_ and NHFO_2_ (median time = 532 s) and at peak exercise. Borg scores are only reported at 2‐min intervals since collected on such timeframe. HR: Heart rate; NHF_air_: Nasal high‐flow without O_2_‐enriched air; NHFO_2_: Nasal high‐flow with O_2_‐enriched air; RR: Respiratory rate; SpO_2_: Oxygen saturation by pulse oximetry; tcPCO_2_: Transcutaneous partial pressure of carbon dioxide; VE: Minute ventilation; VT: Tidal volume.

Physiological and perceptual responses to EET at isotime and peak exercise across experimental conditions are depicted in Table 2 (similar data can be found throughout exercise in Figure 4). For clarity sake, between‐condition differences and 95% confidence intervals are shown in Table 3. Supplemental O_2_ and NHFO_2_ improved SpO_2_ (and tcPCO_2_) vs air and NHF_air_ at isotime (all *p* < 0.001); of note, SpO_2_ was similar between the two former conditions (median ≥ 98%). In general, supplemental O_2_ and NHFO_2_ offered the same physiological benefits, that is, a lower heart rate vs air and NHF_air_ and ventilation vs air (all *p* < 0.001); the latter was mainly driven by lower respiratory rates, though NHFO_2_ lessened tidal volume vs air (*p* = 0.011). NHF_air_ lowered minute ventilation vs air (~9 L·min^−1^; *p* = 0.012); NHFO_2_ reduced minute ventilation vs NHF_air_ (*p* = 0.007) by lowering respiratory rate (*p* = 0.002). NHFO_2_ significantly improved leg discomfort vs air (*p* = 0.028) and NHF_air_ (*p* = 0.010). Some physiological benefits of supplemental O_2_ and NHFO_2_ persisted at peak exercise, including gas exchange parameters vs air and NHF_air_, and lower ventilatory requirements vs air (all *p* ≤ 0.020). Peak‐exercise symptoms across conditions were generally similar except lower dyspnea scores on supplemental O_2_ and NHFO_2_ vs air (*p* = 0.004 and 0.026, respectively).

**TABLE 2:** Physiological and perceptual responses in patients with fibrotic interstitial lung disease at isotime and at the peak of endurance exercise tests across experimental conditions.

	Air	Supplemental O_2_	NHF_air_	NHFO_2_	*p*
Endurance time (s)	346 [247]	683 [903] *†	319 [415]	690 [1338] *†	< 0.001
*Isotime*					
Cardio‐ventilatory					
HR (bpm)	124 ± 12	114 ± 11*†	122 ± 14	111 ± 12*†	< 0.001
VE (L·min^−1^)	62.7 ± 29.1	47.3 ± 21.5*	53.7 ± 22.9 *	44.3 ± 20.0*†	< 0.001
VT (L)	1.51 ± 0.91	1.40 ± 0.76	1.35 ± 0.75	1.26 ± 0.67*	0.016
RR (breaths·min^−1^)	44 ± 10	36 ± 9*†	43 ± 11	37 ± 8*†	< 0.001
Pulmonary gas exchange					
SpO_2_ (%)	87 [17]	98 [2]*†	87 [14]	99 [3]*†	< 0.001
tcPCO_2_ (mmHg)	33 ± 6	37 ± 5*†	32 ± 4	38 ± 7*†	< 0.001
Symptoms					
Dyspnea	7 [3]	4 [3.5]*	6 [1.5]	3.5 [2.5]*†	< 0.001
Leg discomfort	6 [2.5]	5 [3.5]	5.5 [2]	4.5 [3.5]*†	0.009
*Peak exercise*					
Cardio‐ventilatory					
HR (bpm)	125 ± 12	121 ± 12	124 ± 15	119 ± 14*†	0.003
VE (L·min^−1^)	63.7 ± 28.1	50.5 ± 20.7*	56.2 ± 27.0	50.1 ± 20.4*	< 0.001
VT (L)	1.52 ± 0.91	1.35 ± 0.73	1.31 ± 0.75	1.30 ± 0.67*	0.024
RR (breaths·min^−1^)	45 ± 10	40 ± 10*†	46 ± 9	41 ± 9*†	< 0.001
Pulmonary gas exchange					
SpO_2_ (%)	87 [17]	97 [6]*†	86 [15]	98 [5]*†	< 0.001
tcPCO_2_ (mmHg)	33 ± 6	37 ± 6*†	31 ± 4	35 ± 6†	< 0.001
Symptoms					
Dyspnea	7 [1.5]	6 [3.5]*	7 [3]	6.5 [4.5]*	0.048
Leg discomfort	6 [2]	6 [2]	6.5 [2.5]	7 [3.5]	0.914

*Note:* Exercise responses are shown at isotime, that is, at the time corresponding to the end of the shortest experimental condition. Data are also depicted at peak exercise, that is, at the time corresponding to exercise intolerance in each four experimental conditions. Data are mean ± SD or median [interquartile range]. *p* values (right column) refer to main ANOVA or Friedman ANOVA effects. *: *p* < 0.05 vs air; **†**: *p* < 0.05 vs NHF_air_ (post hoc analyses).

Abbreviations: HR: heart rate; NHF_air_: nasal high‐flow without O_2_‐enriched air; NHF_O2_: nasal high‐flow with O_2_‐enriched air; RR: respiratory rate; SpO_2_: oxygen saturation by pulse oximetry; tcPCO_2_: transcutaneous partial pressure of carbon dioxide VE: minute ventilation; VT: tidal volume.

**TABLE 3:** Between‐condition differences and 95% confidence intervals for endurance time and physiological and perceptual responses in patients with fibrotic interstitial lung disease at isotime and at the peak of endurance exercise tests across experimental conditions.

	NHF_air_—Air	O_2_—Air	NHFO_2_—Air	O_2_—NHF_air_	NHFO_2_–NHF_air_	NHFO_2_–O_2_
Endurance time (s)	70 (−8; 192)	328 (203; 750)	584 (254; 876)	288 (139; 718)	422 (243; 782)	70 (−5; 224)
*Isotime*						
Cardio‐ventilatory						
HR (bpm)	‐2 (−7; 3)	‐9 (−15; −4)	−13 (−18; −8)	−7 (−13; −2)	−11 (−16; −6)	−4 (−9; 2)
VE (L·min^−1^)	−9.0 (−16.5; −1.4)	−15.3 (−22.9; −7.8)	−18.4 (−25.9; −10.8)	−6.4 (−13.9; 1.1)	−9.4 (−16.9; 1.9)	−3.0 (−10.5; 4.5)
VT (L)	−0.16 (−0.37; 0.05)	−0.12 (−0.32; 0.09)	−0.25 (−0.45; −0.04)	0.04 (−0.16; 0.25)	−0.09 (−0.30; 0.12)	−0.13 (−0.34; 0.17)
RR (breaths·min^−1^)	‐1 (−6; 3)	−9 (−13; −4)	−7 (−12; −3)	−8 (−12; −3)	−6 (−11; −2)	1 (−3; 6)
Gas exchange						
SpO_2_ (%)	0 (−2; 2)	13 (9; 17)	14 (10; 18)	13 (8; 18)	14 (9; 19)	1 (0; 1)
tcPCO_2_ (mmHg)	−1 (−4; 3)	5 (1; 8)	5 (1; 9)	5 (2; 9)	6 (2; 9)	0 (−3; 4)
Symptoms						
Dyspnea	−0.5 (−2; 0.5)	−2 (−3; −1.5)	−3 (−4; −2)	−1.5 (−3; 0)	−2.5 (−3.5; −0.5)	−1 (−1.5; 0)
Leg discomfort	−0.5 (−1; 1)	−1 (−2; 0)	−2 (−3; −0.5)	−1 (−2; 0)	−1.5 (−2; −0.5)	−0.5 (−1.5; 0.5)
*Peak exercise*						
Cardio‐ventilatory						
HR (bpm)	−1 (−6; 4)	−4 (−9; 0)	−6 (−11; −1)	−3 (−8; 1)	−5 (−10; 0)	−2 (−6; 3)
VE (L·min^−1^)	−7.5 (−16.5; 1.4)	−13.2 (−22.2; −4.3)	−13.6 (−22.6; −4.7)	−5.7 (−14.7; 3.2)	−6.1 (−15.0; 2.9)	−0.4 (−9.3; 8.6)
VT (L)	−0.22 (−0.44; 0.01)	−0.18 (−0.40; 0.05)	−0.23 (−0.47; −0.01)	0.04 (−0.18; 0.27)	−0.01 (−0.23; 0.21)	−0.05 (−0.28; 0.17)
RR (breaths·min^−1^)	0 (−3; 4)	−5 (−9; −2)	−4 (−8; −1)	−6 (−9; −2)	−5 (−8; −1)	1 (−3; 5)
Gas exchange						
SpO_2_ (%)	0 (−2; 2)	12 (9; 17)	14 (8; 17)	12 (8; 17)	13 (9; 18)	1 (0; 2)
tcPCO_2_ (mmHg)	−1 (−4; 2)	4 (1; 7)	3 (−1; 6)	5 (2; 8)	4 (1; 7)	−1 (−5; 2)
Symptoms						
Dyspnea	−0.5 (−1.5; 0.5)	−1 (−2; −0.5)	−1.5 (−2.5; 0)	−0.5 (−2; 0.5)	−1 (−2.5; 1)	0 (−1.5; 1)
Leg discomfort	0 (−1; 0.5)	0 (−1; 1)	0 (−1; 1.5)	0 (−1; 1)	0 (−1.5; 1.5)	0 (−0.5; 1)

*Note:* Exercise responses are shown at isotime, that is, at the time corresponding to the end of the shortest experimental condition. Data are also depicted at peak exercise, that is, at the time corresponding to exercise intolerance in each four experimental conditions. Bonferroni or dependent‐sample Hodges‐Lehman Median Difference tests were applied to determine between‐condition differences and 95% confidence intervals for normally distributed continuous variables and non‐normally distributed continuous and discrete variables, respectively.

Abbreviations: HR: heart rate; NHF_air_: nasal high‐flow without O_2_‐enriched air; NHFO_2_: nasal high‐flow with O_2_‐enriched air; RR: respiratory rate; SpO_2_: oxygen saturation by pulse oximetry; tcPCO_2_: transcutaneous partial pressure of carbon dioxide; VE: minute ventilation; VT: tidal volume.

### 3.4 Vastus Lateralis Muscle Oxygenation

**FIGURE 5:**
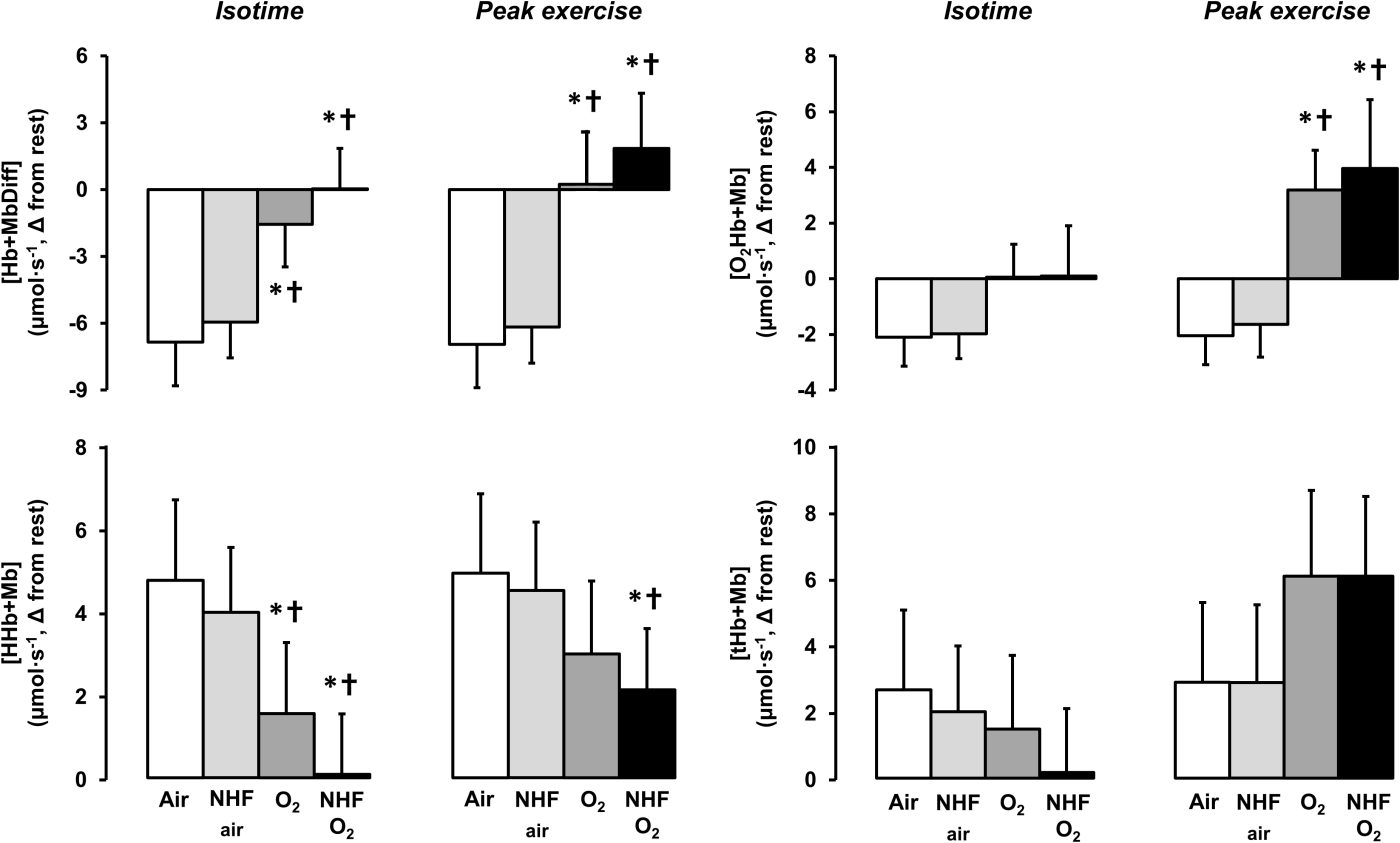
Changes in *vastus lateralis* concentrations of oxy—deoxyhemoglobin and myoglobin difference, oxyhemoglobin and myoglobin, deoxyhemoglobin and myoglobin, and total hemo‐ and myoglobin by near‐infrared spectroscopy in fibrotic interstitial lung disease across experimental conditions during endurance exercise test. *: *p* < 0.05 vs air; †: *p* < 0.05 vs NHF_air_ (post hoc analyses). Hb + MbDiff: Oxy‐deoxyhemoglobin and myoglobin difference; HHb + Mb: deoxyhemoglobin and myoglobin; NHF_air_: nasal high‐flow without O_2_‐enriched air; NHFO_2_: nasal high‐flow with O_2_‐enriched air; O_2_: oxygen supplementation; O_2_Hb + Mb: oxyhemoglobin and myoglobin; tHb + Mb: total hemo‐ and myoglobin.

Figure 5 depicts the consistent effects of supplemental O_2_ and NHFO_2_ in improving *quadriceps* muscle oxygenation during EET. Specifically, both interventions improved [Hb + MbDiff] vs air and NHF_air_ at isotime and peak exercise (condition × exercise time interaction, *F* = 12.4, *p <* 0.001). This was achieved by concomitant increase in [O_2_Hb + Mb] (condition × exercise time interaction, *F* = 10.1, *p <* 0.001) and decline in [HHb + Mb] (condition × exercise time interaction, *F* = 5.9, *p <* 0.001). We found a condition × exercise time interaction (*F* = 4.3, *p <* 0.001) in [tHb + Mb] but post hoc analyses did not reach significance (*p ≥* 0.096). Muscle oxygenation did not differ between air versus NHF_air_ and supplemental O_2_ versus NHFO_2_ (all *p* > 0.05). Table S1 depicts between‐condition differences and 95% confidence intervals.

## 4 Discussion

This pilot study explores whether NHF_air_ and supplemental O_2_ would offer (i) independent and (ii) synergistic (with NHFO_2_ vs supplemental O_2_) beneficial effects on dyspnea and exercise tolerance in *f*‐ILD. Contrary to our hypotheses, our main findings indicate that (i) unlike supplemental O_2_, NHF_air_ did not significantly improve dyspnea and exercise tolerance vs air, though estimates came with uncertainty; (ii) supplemental O_2_ and NHFO_2_ provided similar improvements in these outcomes at “iso‐O_2_ saturation” and; (iii) supplemental O_2_ and NHFO_2_ share common physiological benefits such as reduced minute ventilation and increased *quadriceps* muscle oxygenation. Our findings thus provide original evidence that physiological benefits derived from O_2_ supplementation are presumably the primary drivers of dyspnea relief and improved exercise tolerance on NHFO_2_ vs air in *f*‐ILD, with no additional benefit of NHF_air_. These findings will, however, require further investigation with adequate (i.e., larger) sample size in line with the number of experimental conditions being compared.

Previous research has investigated the benefits of NHFO_2_ in reversing exertional hypoxemia, lessening dyspnea and improving patients' exercise capacity in *f*‐ILD [13, 20, 21, 22, 41]. Our study innovates by specifically comparing four distinct experimental conditions (of which “iso‐O_2_ saturation” between supplemental O_2_ and NHFO_2_); this unique design allowed us to “isolate” the physiological and perceptual effects of respiratory support from improved oxygenation in patients with *f*‐ILD and severe activity‐related hypoxemia.

Despite apparent methodological differences, all above‐mentioned studies that compared room air and NHFO_2_ consistently reported symptomatic relief (i.e., isotime dyspnea) and longer exercise endurance time under the latter in *f*‐ILD [13, 20, 21]. However, whether NHFO_2_ provides superior benefits vs O_2_ therapy remains debated. In fact, supplemental O_2_ delivered at low flow rates (4 L·min^−1^ via nasal cannula) has been shown inappropriate to fully correct hypoxemia in *f*‐ILD [13]; greater dyspnea alleviation and exercise tolerance on NHFO_2_ may therefore reflect a larger improvement in systemic oxygenation and/or a specific effect of NHF‐related respiratory support but does not allow teasing out any separate contribution in this study [13]. In three studies, supplemental O_2_ delivered via a Venturi mask yielded either comparable [21] or lower [20, 22] exercise tolerance vs NHFO_2_, together with similar isotime dyspnea ratings (when available [20, 21]) in *f*‐ILD. Of note, this study shows that, when (i) O_2_ desaturation is appropriately corrected (i.e., O_2_ saturation > 94%, defining mild hypoxemia [42], herein ~97% at end‐exercise) and, (ii) O_2_ saturation is comparable between O_2_ therapy systems, supplemental O_2_ and NHFO_2_ offered similar benefits on dyspnea relief and exercise tolerance in these patients (with trivial effect for the latter outcome though this came with uncertainty as estimates ranged from no to worthwhile effects). This was not evident in other investigations since either O_2_ saturation (i) was ~90% [21, 22] or, (ii) differed between modalities with medium effect size favouring NHFO_2_ [20].

Different aspects of our research, however, require specific attention or caution. First, a limited proportion of our participants (56%) showed an endurance time on room air (i.e., our “control” condition) falling within the “desirable” time limit range (180–480 s) [35]. Although such fraction corresponds to what is typically observed in large samples of respiratory patients, this may impede the responsiveness of endurance time to interventions, considering the power‐duration relationship [35]. Performing multiple EETs in the “control” condition to first establish individualized intensities to obtain the targeted duration was not feasible or realistic because: (i) the order of the 4 experimental conditions would not have been randomized anymore (as per our study design) and, (ii) we also aimed to limit the number of experimental visits considering patients' frailty and severity in a study that already required 5 CPETs. Second, and for similar reason**s**, our study design (leading to an endurance time > 480 s in most patients on O_2_ together with an imposed maximum duration of 30 min) was sub‐optimal to fully address the comparison between O_2_ and NHFO_2_ for exercise tolerance. This would have required O_2_ to be considered the “control” condition, again targeting exercise duration between 180 and 480 s, which was not the case in the present study.

We hypothesized that NHF_air_ would lessen dyspnea and improve exercise tolerance vs air in *f‐*ILD due to independent physiological benefits (detailed in *Introduction* and dedicated section *below*). Yanagita et al. previously demonstrated that NHF_air_ did not significantly improved endurance time, though they suggested the possibility of clinical relevance vs air in these patients [41]. We, however, anticipated different results since our sample presented with more advanced lung restriction (forced vital capacity ~70 vs. 85% predicted) and gas exchange abnormalities (lung diffusing capacity for carbon monoxide ~30 vs. 70% predicted). In this context, the physiological benefits of NHF_air_ (e.g., reduced work of breathing or improved gas exchange efficiency [23, 24]) might have offered greater improvement in these outcomes in patients showing poorer pulmonary compliance and gas exchange [43]. Our results do not exclude that NHF_air_ may meaningfully improve exercise capacity since the upper bound of the estimate (192 s) exceeds the MCID. It is also paramount to highlight that NHF_air_ typically elicits large inter‐individual differences on endurance time [39, 44] suggesting it might be used on an individual basis in patients with *f*‐ILD: 6 subjects exceeded the MCID of 105 s or 33% [35], but 3 concomitantly showed decline above these values on NHF_air_ vs air. Altogether, the present study holds high external validity as 88% of patients showed severe exertional hypoxemia during incremental exercise testing (SpO_2_ ≤ 88%, the threshold at which ambulatory O_2_ therapy may be prescribed in *f*‐ILD [6]; mean SpO_2_ = 80%). This was not the case in the above‐mentioned investigation as patients had nadir SpO_2_ ~ 92% [41], and therefore, NHFO_2_ would not be used in clinical practice.

Our study reports breathing pattern during exercise with NHFO_2_ in *f*‐ILD, including relevant isotime measurements across experimental conditions. We observed that O_2_ supplementation and NHFO_2_ offered similar reduction in isotime minute ventilation (~15–18 L·min^−1^ or ~25%–30%) vs room air, primarily by lowering respiratory rate (~8 breaths·min^−1^). Of note, this magnitude corroborates with what we [8] and others [9, 45] reported on supplemental O_2_ using a standard metabolic cart, giving credit to our measurements by respiratory inductance plethysmography. Schaeffer et al. [9] showed that dyspnea intensity on room air was strongly associated with an increased inspiratory neural drive (using diaphragmatic electromyography), both improving on supplemental O_2_ in *f*‐ILD due to a more harmonious neuromechanical coupling of the respiratory system. Such mechanisms are, therefore, likely crucial for dyspnea relief and, subsequently, improved exercise tolerance on NHFO_2_ in this patient population. NHF_air_ also led to a~9 L·min^−1^ lower isotime minute ventilation; such reduction is thought to originate from the washout of the anatomical dead space preventing rebreathing of expired gas, overall improving alveolar ventilation [23, 46]. However, NHF_air_ did not address issues of critical hypoxemia (mean SpO_2_ = 83%), a hallmark of *f*‐ILD and an important source of increased inspiratory neural drive [3, 47, 48]. It is, therefore, conceivable that the beneficial effects of NHF_air_ on ventilatory drive and total ventilation were outperformed by those from supplemental O_2_ (~twice as large), the net result being no change in dyspnea and exercise tolerance on NHF_air_ versus air.

We recently demonstrated that severe exertional hypoxemia results in impaired muscle O_2_ delivery and, consequently, heightened fatigue in *f*‐ILD [8]. Accordingly, we showed that, on supplemental O_2_, *quadriceps* muscle oxyhemoglobin concentration and twitch force decline to magnetic nerve stimulation no longer differed from healthy controls, suggesting that improving O_2_ delivery and, in turn, lessening contractile fatigue is pivotal to enhance exercise capacity in these patients [8]. As stated before, these laboratory findings, however, cannot be easily translated into practice due to the gas delivery system used (a Douglas reservoir bag connected to a two‐way non‐rebreathing valve) [10]. The present results indicate that such effects may be readily achieved on NHFO_2_, as shown by greater oxy‐deoxyhemoglobin concentration difference by NIRS. Of note, NHFO_2_ may also improve muscle oxidative metabolism in *f*‐ILD [7], thus delaying metabolic acidosis with potential positive consequences on ventilatory drive and dyspnea [3]. Such mechanisms (e.g., lessened minute ventilation or improved muscle O_2_ availability) are also main contributors to increased exercise tolerance on supplemental O_2_ and NHFO_2_ versus NHF_air_ in severely‐hypoxemic *f*‐ILD. Altogether, NHFO_2_ therapy may have various systemic effects in *f*‐ILD: teasing out the contribution of the different systems in improving exercise tolerance in these patients remains to be examined [49].

Our sample size calculation was based on the average improvement in exercise time on NHFO_2_ vs room air (i.e., 2 conditions) during an EET in *f*‐ILD. Consequently, we acknowledge our study may be under‐powered for comparison between other experimental conditions. A ~2‐min lag time between tcPCO_2_ and actual partial pressure of arterial CO_2_ (PaCO_2_) has been reported [50]. However, tcPCO_2_ is the best on‐exercise surrogate of PaCO_2_ in chronic obstructive pulmonary disease, another lung condition characterized by gas exchange abnormalities [51]. Also, we report a**~**5 (from ~33 to ~37–38) mmHg increase in isotime tcPCO_2_ on supplemental O_2_ and NHFO_2_ vs room air due to lessened (~25%–30%) excess ventilation, being concordant with studies on supplemental O_2_ in *f*‐ILD [8, 9]. We are therefore confident that our tcPCO_2_ values on air truly reflect excess ventilation during exercise in *f*‐ILD [4] when those on supplemental O_2_ and NHFO_2_ reflect lower ventilatory demand (owing to lessened ventilatory drive [9]). Although the sequence of experimental conditions was randomized, participants could not be blind to the gas delivery system being used (face mask vs NHFO_2_). Crucially, however, patients remained blind to gas mixture and SpO_2_ readings. We also acknowledge that we did not apply a balanced‐order sequence for randomization, which likely introduced bias as, for instance, room air and NHF_air_ conditions were performed were on 8 and 5 occasions, respectively. Walking may have been a more relevant testing modality for patients in daily life. In fact, we previously found that 50% of patients showed severe hypoxemia during a 6‐min walk test but not during cycling [8]. Thus, the positive effects of O_2_ supplementation we herein report may be even more relevant during walking in *f*‐ILD, keeping in mind that new high‐flow systems can function on battery but their use is likely restricted to in‐patient pulmonary rehabilitation. Moreover, we largely focused on patients with severe hypoxemia as SpO_2_ ≤ 88% is the usual threshold for the use of supplemental O_2_ during exercise in *f*‐ILD. We reasoned that focusing on this population would enhance the external validity of our results. The present physiological study offers incomplete insights on the mechanisms of dyspnea relief and improved exercise tolerance with NHFO_2_ in *f*‐ILD; future research may provide more comprehensive assessment of, for instance, inspiratory neural drive and skeletal muscle fatigability which may be explored from diaphragmatic electromyography and peripheral nerve stimulation [9, 52].

In conclusion, our study reports that both acute O_2_ supplementation and NHFO_2_ provided improvements in exertional dyspnea and exercise tolerance at “iso‐O_2_ saturation” in severely‐hypoxemic patients with *f*‐ILD, primarily due to alike decline in ventilatory requirements. Our study thus suggests that physiological benefits derived from supplemental O_2_ are presumably the primary drivers of dyspnea relief and improved exercise tolerance on NHFO_2_ (vs. air) in *f*‐ILD, with no added benefit of NHF_air_. Yet, further research is required to confirm these preliminary findings with adequate (i.e., larger) sample size due to the number of experimental conditions we compared.

## Author Contributions

**Sarah Thivent:** methodology, software, data curation, investigation, validation, formal analysis, funding acquisition, visualization, project administration, resources, writing – original draft. **Marylise Ginoux:** investigation, validation, supervision, project administration, visualization, writing – review and editing. **Samuel Verges:** conceptualization, methodology, investigation, validation, supervision, funding acquisition, visualization, resources, writing – review and editing, project administration. **Frédéric Hérengt:** conceptualization, methodology, investigation, validation, supervision, funding acquisition, visualization, project administration, resources, writing – review and editing. **Mathieu Marillier:** conceptualization, methodology, software, investigation, validation, formal analysis, supervision, funding acquisition, visualization, project administration, resources, writing – original draft.

## Funding

This work was supported by Fonds de dotation Agir Pour les Maladies Chroniques (AO 2022) and Fisher and Paykel Healthcare.

## Ethics Statement

This study was registered at ClinicalTrials.gov (trial registration: NCT07129707); however, the trial registration omits some physiological (e.g., heart rate) or perceptual (e.g., leg discomfort) variables or time‐point (peak exercise data) of interest we herein report in the manuscript. This study was approved by an independent ethics board (CPP SUD‐OUEST and OUTRE MER III, IDRCB: 2022‐A00774‐39) and registered at clinicaltrials.gov (NCT07129707). Written informed consent was obtained from all participants before study enrollment.

## Conflicts of Interest

The authors declare no conflicts of interest.

## Supporting information


**Table S1:** Between‐condition differences and 95% confidence intervals for *Vastus lateralis* muscle oxygenation by near‐infrared spectroscopy in patients with fibrotic interstitial lung disease at rest, isotime and at the peak of endurance exercise tests across experimental conditions.

## Data Availability

The data that support the findings of this study are available from the corresponding author upon reasonable request.
