# Supplementary material for: Determinants of Improved Dyspnea and Exercise Tolerance With Nasal High‐Flow O2 Therapy in Fibrotic Interstitial Lung Disease: A Pilot Physiological Study
Source: Respirology. 2026 Apr 9;31(7):732–44. doi: 10.1002/resp.70251 (PMC13342421; doi:10.1002/resp.70251)
Supplement: Supplementary file 1 — Table S1: Between‐condition differences and 95% confidence intervals for Vastus lateralis muscle oxygenation by near‐infrared spectroscopy in patients with fibrotic interstitial lung disease at rest, isotime and at the peak of endurance exercise tests across experimental conditions. [file RESP-31-732-s001.docx]

***Supplementary information***

**Determinants of improved dyspnea and exercise tolerance with nasal high-flow O_2_ therapy in fibrotic interstitial lung disease: a pilot physiological study**

Sarah Thivent, Marylise Ginoux, Samuel Vergès, Frédéric Hérengt and Mathieu Marillier

**MATERIALS AND METHODS**

**Near-infrared spectroscopy**

Near-infrared spectroscopy is a non-invasive, optical technique that relies on the use of infrared wavelength light (650-950 nm). Briefly, photons in the near-infrared spectrum are able to cross tissue over several cm^1^. Near-infrared light illuminates the body tissue under investigation and is reflected back to receptors. In this context, the difference between the intensity of the light emitted and received provides information on the concentration of the different chromophores within a tissue at a given wavelength. Consequently, when the intensity of the light received at the receptors is low, the absorption of infrared photons is high and, therefore, indicates that the concentration of the chromophore in question is also high^2^. Near-infrared spectroscopy usually allows to determine the concentration of 2 main chromophores (oxy- and deoxyhemoglobin), while total hemoglobin is calculated as the sum of oxy- + deoxyhemoglobin concentration^3, 4^, the latter reflecting changes in tissue blood volume within the illuminated area^5, 6^. Near-infrared spectroscopy parameters reflect the dynamic balance between O_2_ demand and supply in the tissue micro-circulation: oxy- and total hemoglobin concentrations are mostly sensitive to blood flow and O_2_ delivery while deoxyhemoglobin concentration is closely associated with changes in venous O_2_ content and therefore tissue O_2_ extraction^3, 7^. Oxy-deoxyhemoglobin concentration difference is calculated as oxyhemoglobin – deoxyhemoglobin difference and is an estimate of change in tissue oxygenation^8^.

**RESULTS**

**Vastus lateralis muscle oxygenation**

**Table S1** describes between-condition differences and 95% confidence intervals for *vastus lateralis* muscle oxygenation during endurance exercise test across experimental conditions. These results complement information provided in the main text (see *Results*) and data shown in **Figure 4** (mean ± SD and ANOVA condition × exercise time interaction with corresponding post-hoc analyses).

**Table S1.** Between-condition differences and 95% confidence intervals for *vastus lateralis* muscle oxygenation by near-infrared spectroscopy in patients with fibrotic interstitial lung disease at rest, isotime and at the peak of endurance exercise tests across experimental conditions.

|  | **NHF_air_ – Air** | **O_2_ – Air** | **NHFO_2_ – Air** | **O_2_ – NHF_air_** | **NHFO_2_ – NHF_air_** | **NHFO_2_ – O_2_** |
| --- | --- | --- | --- | --- | --- | --- |
| ***Rest*** |  |  |  |  |  |  |
| Hb+MbDiff (μmol·s^-1^) | -0.04 (-3.39; 3.31) | -0.06 (-3.40; 3.29) | -0.33 (-3.68; 3.02) | -0.02 (-3.37; 3.33) | -0.30 (-3.65; 3.05) | -0.28 (-3.63; 3.07) |
| O_2_Hb+Mb (μmol·s^-1^) | -0.03 (-2.56; 2.50) | -0.04 (-2.56; 2.49) | -0.25 (-2.78; 2.28) | 0.00 (-2.53; 2.53) | -0.22 (-2.75; 2.31) | -0.22 (-2.75; 2.31) |
| HHb+Mb (μmol·s^-1^) | 0.05 (-2.17; 2.28) | 0.01 (-2.22; 2.24) | 0.08 (-2.15; 2.30) | -0.05 (-2.27; 2.18) | 0.02 (-2.20; 2.25) | 0.07 (-2.16; 2.30) |
| tHb+Mb (μmol·s^-1^) | 0.02 (-3.39; 3.42) | 0.00 (-3.41; 3.41) | -0.18 (-3.59; 3.23) | -0.02 (-3.43; 3.39) | -0.20 (-3.61; 3.21) | -0.18 (-3.59; 3.23) |
| ***Isotime*** |  |  |  |  |  |  |
| Hb+MbDiff (μmol·s^-1^) | 0.88 (-2.46; 4.24) | 5.29 (1.94; 8.64) | 6.88 (3.53; 10.23) | 4.40 (1.05; 7.75) | 5.99 (2.64; 9.34) | 1.59 (-1.76; 4.94) |
| O_2_Hb+Mb (μmol·s^-1^) | 0.12 (-2.41; 2.65) | 2.16 (-0.37; 4.69) | 2.19 (0.34; 4.72) | 2.04 (-0.49; 4.57) | 2.07 (-0.45; 4.60) | 0.03 (-2.50; 2.56) |
| HHb+Mb (μmol·s^-1^) | -0.77 (-3.00; 1.45) | -3.20 (-5.43; -0.98) | -4.67 (-6.90; -2.45) | -2.43 (-4.66; -0.20) | -3.90 (-6.13; -1.67) | -1.47 (-3.69; 0.76) |
| tHb+Mb (μmol·s^-1^) | -0.66 (-4.06; 2.75) | -1.17 (-4.58; 2.23) | -2.48 (-5.89; 0.93) | -0.52 (-3.93; 2.89) | -1.82 (-5.23; 1.58) | -1.31 (-4.72; 2.10) |
| ***Peak exercise*** |  |  |  |  |  |  |
| Hb+MbDiff (μmol·s^-1^) | 0.78 (-2.57; 4.14) | 7.19 (3.84; 10.54) | 8.81 (5.45; 12.16) | 6.40 (3.05; 9.75) | 8.02 (4.67; 11.37) | 1.62 (-1.73; 4.97) |
| O_2_Hb+Mb (μmol·s^-1^) | 0.40 (-2.13; 2.93) | 5.23 (2.70; 7.76) | 5.99 (3.47; 8.53) | 4.83 (2.31; 7.36) | 5.60 (3.07; 8.13) | 0.77 (-1.76; 3.29) |
| HHb+Mb (μmol·s^-1^) | -0.42 (-2.65; 1.81) | -1.95 (-4.17; 0.28) | -2.80 (-5.04; 0.58) | -1.53 (-3.75; 0.70) | -2.39 (-4.62; -0.16) | -0.86 (-3.09; 1.36) |
| tHb+Mb (μmol·s^-1^) | -0.02 (-3.43; 3.39) | 3.18 (-0.23; 6.59) | 3.19 (-0.22; 6.60) | 3.20 (-0.20; 6.61) | 3.21 (-0.20; 6.62) | 0.01 (-3.40; 3.41) |

Data were averaged over the last 30 s of the resting period (baseline) to determine isotime and peak-exercise values as Δ from rest as typically done by near-infrared spectroscopy. Exercise responses are shown at isotime, that is, at the time corresponding to the end of the shortest experimental condition. Data are also depicted at peak exercise, that is, at the time corresponding to exercise intolerance in each four experimental conditions.

Bonferroni post-hoc tests were applied to determine between-condition differences and 95% confidence intervals.

*Definition of abbreviations*: Hb+MbDiff: oxy-deoxyhemoglobin and myoglobin difference; HHb+Mb: deoxyhemoglobin and myoglobin; NHF_air_: nasal high-flow without O_2_-enriched air; NHFO_2_: nasal high-flow with O_2_-enriched air; O_2_: oxygen supplementation; O_2_Hb+Mb: oxyhemoglobin and myoglobin; tHb+Mb: total hemo- and myoglobin.

**REFERENCES**

1 Perrey S. Non-invasive NIR spectroscopy of human brain function during exercise. Methods. 2008; **45**: 289-99.

2 Wahr JA, Tremper KK, Samra S, Delpy DT. Near-infrared spectroscopy: theory and applications. J Cardiothorac Vasc Anesth. 1996; **10**: 406-18.

3 Ferrari M, Mottola L, Quaresima V. Principles, techniques, and limitations of near infrared spectroscopy. Can J Appl Physiol. 2004; **29**: 463-87.

4 Ferrari M, Quaresima V. A brief review on the history of human functional near-infrared spectroscopy (fNIRS) development and fields of application. Neuroimage. 2012; **63**: 921-35.

5 Hoshi Y, Kobayashi N, Tamura M. Interpretation of near-infrared spectroscopy signals: a study with a newly developed perfused rat brain model. J Appl Physiol (1985). 2001; **90**: 1657-62.

6 Van Beekvelt MC, Colier WN, Wevers RA, Van Engelen BG. Performance of near-infrared spectroscopy in measuring local O(2) consumption and blood flow in skeletal muscle. J Appl Physiol (1985). 2001; **90**: 511-9.

7 Rolfe P. In vivo near-infrared spectroscopy. Annu Rev Biomed Eng. 2000; **2**: 715-54.

8 Rupp T, Perrey S. Prefrontal cortex oxygenation and neuromuscular responses to exhaustive exercise. Eur J Appl Physiol. 2008; **102**: 153-63.
